# The Role of Parenting Styles and Empathy in the Perpetration of Cyberbullying in a Sample of Adolescents

**DOI:** 10.3390/children13081015

**Published:** 2026-07-30

**Authors:** Adriana Martins, Margarida Simões, Ana Paula Monteiro, Catarina Pinheiro Mota, Ana Isabel Sani, Inês Carvalho Relva

**Affiliations:** 1 Department of Education and Psychology, University of Trás-os-Montes and Alto Douro, 5000-801 Vila Real, Portugal; margaridas@utad.pt (M.S.); catppmota@utad.pt (C.P.M.); irelva@utad.pt (I.C.R.); 2 Centre for Research and Intervention in Education (CIIE), University of Porto, Rua Alfredo Allen, 4200-135 Porto, Portugal; 3 Center in Sports Sciences, Health Sciences and Human Development (CIDESD), 5000-800 Vila Real, Portugal; 4 RISE Health, University of Trás-os-Montes and Alto Douro, 5000-801 Vila Real, Portugal; 5 Center of Psychology, University of Porto (CPUP), Rua Alfredo Allen, 4200-135 Porto, Portugal; 6 Research Center on Child Studies (CIEC), University of Minho, 4710-057 Braga, Portugal; anasani@ufp.edu.pt; 7 Faculty of Human and Social Sciences, University Fernando Pessoa (UFP), Praça 9 de Abril, 349, 4249-004 Porto, Portugal

**Keywords:** cyberbullying, parenting styles, empathy, adolescents, intervention

## Abstract

**Background:** The study of cyberbullying has been growing worldwide; however, the literature is still scarce regarding risk and protection factors. **Objectives**: Therefore, the main objective of the present study was to analyze cyberbullying from the perspective of the aggressors and their association with authoritative and authoritarian parenting styles and with the two components of empathy. **Methods**: The sample consisted of 422 participants, students of the 2nd and 3rd cycles in schools and Secondary Education in the North region of Portugal, aged 12 to 17 years. For data collection, the Sociodemographic Questionnaire, the Cyberbullying Questionnaire (CBQ), the Parenting Styles & Dimensions Questionnaire: Short Version (PSDQ), and the Short Version of the Basic Empathy Scale (BES-A) were used. **Results**: It was found that positive associations were identified between cyberbullying behaviours and authoritarian style in the father figure and the mother figure. Low positive associations were also identified between the father figure’s authoritative parenting style and cognitive empathy, and moderate positive associations between the mother figure’s authoritative parenting style and cognitive empathy. There were differences between males and females in both empathy components, with females showing higher levels of affective and cognitive empathy than males. Regarding parental styles, the authoritarian style of the father figure stood out as the only predictor of cyberbullying behaviors. **Conclusions**: It is considered important to educate parents towards healthier family functioning and encourage the abandonment of punitive discipline to promote a reduction in the perpetration of cyberbullying and an increase in empathy levels in the younger generation. It will also be important to raise awareness among adolescents about the consequences of internet misuse.

## 1. Introduction

Parenting has faced many challenges today, with parents finding it increasingly difficult and stressful to educate their children [1]. During adolescence, young people struggle for autonomy [2], which can often pose significant difficulty for many parents [3]. Therefore, as stress levels increase, parents may be more likely to adopt inappropriate practices with their children [4]. Affective bonding, open communication, and parental control in the face of adolescent behaviors are key factors in combating the inappropriate behavior of young people (e.g., cyberbullying) [5]. In families where interaction between parents and children is low, punishment is excessive, and supervision is scarce, young people are highly likely to engage in aggressive behaviour [6].

Currently, electronic devices with internet access are recognized as powerful communication and leisure tools for diverse population groups, particularly among adolescents [7]. In 2022, 100% of young people aged 16–24 used the Internet [8]. It is worth noting that the media have invaded individual interactions, significantly changing how [9], making it very difficult today to live without being connected to the internet [10]. Social life has undergone significant changes, and it is very easy to share information and knowledge with the rest of the world [9]. The internet can offer various benefits to its users [11], such as shopping, handling banking issues, and taking classes without leaving home [12]. However, some users may use it for improper purposes, which is worrying [13]. Constant exposure to new technologies can put users’ safety and emotional and psychological well-being [14]. During adolescence, the search for and development of identity play a significant role in internet use, and adolescents seek immediate gratification through online communication, which often leads them to lose control over what they share online, potentially exposing them to risks such as cyberbullying [15].

Humans are born with neural circuits for affection and gradually develop the ability to grasp others’ emotional states and respond empathetically [16]. Therefore, for there to be a healthy interaction and mutual trust between two individuals, it is strictly necessary that both develop empathy [16]. This construct has received special attention in psychological practice, as it is closely related to expected moral and social functioning [17]. Empathy promotes increased social connection [18], greater emotional well-being [19], and better overall health [20]. While individuals with low empathic capacity tend to develop more antisocial and aggressive behaviours, those with high empathy are more predisposed to adopt prosocial behaviours [21].

### 1.1. Cyberbullying

Cyberbullying is considered a major risk resulting from negative experiences using new technologies to harm others [14,22]. In addition, it is characterized as violent, repeated, and intentional behavior in which electronic devices are used to hit each other to harm them [23,24]. This phenomenon occurs through digital means, channels, involving insults and humiliations sent via email, messages, websites, or other means, and has become a concern due to its impact on adolescents [25,26,27]. In the context of cyberbullying, it is possible to identify different actors, namely the victim, the aggressor or cyberbully, who uses the Internet to perpetrate aggressive behavior against the victim, the bystander and individuals who simultaneously assume the role of cyberbully and victim [28]. Cyberbullying, compared to traditional bullying, is more complex [29] and can have more significant consequences, as it can reach a wider audience, be practiced anonymously, and occur at any time of the day [30,31,32]. The prevalence of cyberbullying is difficult to quantify accurately due to the diversity of methodologies used in studies, particularly in data collection methods, research designs, and sample characteristics [28]. Despite these limitations, the report by the United Nations Children’s Fund [33], based on a sample of over 170,000 young people aged between 13 and 24 from 30 countries, revealed that one in three participants had been a victim of cyberbullying. The same report also showed that one in five children had missed school because of this form of violence.

In Portugal, the results of the study ‘Cyberbullying among young people in Portugal: Predictors of Victimisation and Perpetration’, obtained through a national survey conducted on a representative sample of 1262 young people aged between 11 and 21, of whom 50.4 per cent were male, indicated that one in 12 participants reported having engaged in cyberbullying behaviour with high frequency. Among the most frequently reported behaviours were the disclosure of the victim’s private information, harassment and the posting of offensive or malicious comments [34]. Prevalence of cyberbullying practices is most significant in adolescence and is characterized as a period of great vulnerability [35,36].

It is possible to distinguish direct online attacks, i.e., verbal, and social attacks directed at the victim, with the intention of disturbing, from indirect attacks, in which the aggressors pretend to be another person, manipulate content and hack, changing the password to make it impossible for the individual to access the personal accounts [37,38]. Dissemination of content produces a digital fingerprint, which can be seen by many people [39,40], and sometimes it can be difficult to remove information from social networks [41]. Cyberbullying incidents, such as publishing inappropriate photographs, can be quickly and easily disclosed and reach a potentially large audience [29].

Mobile phones, tablets, computers, internet-connected watches, and video game consoles are the most commonly used devices for cyberbullying [40]. Online games, mostly used by school-age young males, allow their users to talk through headphones, and this is a common way of spreading cyberbullying practices [42].

In cyberbullying, there are three types of actors, namely observers, victims, and perpetrators [43]. The latter act anonymously, having the possibility to hide their identity, using false names on social networks, which corresponds to an advantageous factor for adolescents to engage in cyberbullying behaviors, since it is difficult to identify them [44]. The low level of parental supervision, the perception that attacks will not be reported by victims, the lack of consequences for behavior and the low level of intervention by observers are major contributors to the spread of the phenomenon [42]. According to the literature, there are a few risk factors for the perpetration of cyberbullying, such as easy access to new media, anxiety, moral disinterest, low levels of self-esteem, impulsiveness, victimization and perpetration of traditional bullying [32,42,45].

### 1.2. Parental Styles

As regards parental performance, there are considerable differences in knowledge of child development, resilience and emotional regulation capacities, and the educational practices adopted by parents [46]. The main problems related to parental styles include the discipline parents provide, the degree of supervision, and the quality and sufficiency of the care they provide to their descendants [47].

The parental socialization model developed by Maccoby and Martin [48] is based on two autonomous dimensions, namely responsiveness and control, the first of which refers to the way parents respond to the needs of their children, while the second concerns the way they apply parental requirements to regularize the behavior of young people. Both are used to classify different parental styles, and in each case, different practices, values, attitudes, and behaviours are adopted [49]. According to Baumrind [50], three parental styles can be identified: permissive, authoritarian, and authoritative. Regarding the former, parents set few limits and are reluctant to enforce obligations, i.e., there is no control or restriction on the child’s behavior [50,51]. In general, children of permissive parents tend to be impulsive, undisciplined, and have little control over their behavior [52]. As for the authoritarian style, it is characterized by significant rigidity and intolerance [50], in which parents are perceived as unwarm, unresponsive, and quite punitive [51]. Children who belong to authoritarian families are more likely to be introverted, depressed and anxious [52]. As for the authoritative style, according to Baumrind [50], it seeks to exploit the child’s hidden abilities, emphasizing positive reinforcement and avoiding punishment as the main means of control. Therefore, it corresponds to a more balanced parental style, with the main objective of helping the child perceive the negative consequences of his behaviours, while allowing him to explore the world with established limits to develop his self-control [52]. Later, a fourth parental style, designated the negligent style [53], emerged. In this, parents show little involvement and concern for their children’s needs and experiences [54], contributing to the development of low social and academic skills in young people [52].

The primary function of parenting is to enable the individual to construct his own sense of integrity while confronting others’ impositions [53]. Parents play a central role in their children’s emotional understanding and regulation. Still, when this task is poorly performed, it may be a risk factor for aggressive behaviour in younger children [55].

### 1.3. Empathy

One of the great abilities of human beings is social interaction, which, to be effective, requires an understanding of other people’s emotions, thoughts, behaviours, and desires [56]. Empathy can be seen as an important psychological process that makes us social beings, able to promote adaptive outcomes, such as increased emotional well-being and enhancement of social connection, from which we can understand our desires and predict the behavior of other individuals, which may contribute to a more harmonious interaction [19,57]. The term empathy refers metaphorically to the emotional capacity to understand another person’s feelings [58], communicating that understanding and helping them feel understood [59]. The empathic potential contributes to rapid approximation of the other’s emotional states, allowing for greater cooperation and coordination with the same [60]. Therefore, this construct translates into a social competence of projection that develops through peer contact [61]. According to Bošnjaković and Radionov [57], empathy is the basis of morality, influencing prosocial behaviour, compassion, and care for others, thereby inhibiting aggressive and hostile behaviour. Batson et al. [62] noted that “empathic emotions activate an altruistic motivation to benefit the specific individual(s) for whom one feels empathy” (p. 629).

The concept of empathy can be understood in terms of two primary constructs [63]: affective empathy, which is natural and based on a low-level process, and cognitive empathy, which requires greater deliberation and involves high-level processes [64]. The first occurs when there is the ability to truly experience and share the other’s emotional states [65], which can arouse closeness and concern toward him [66]. In affective empathy, there is recognition of the other’s emotions based on their facial expressions. These tones of voice and body movements activate the emotional response to the situation the person is facing and facilitate the accurate identification of the person’s emotional state [67]. On the other hand, cognitive empathy refers to the ability to intellectually identify and understand another person’s subjectivity and feelings without experiencing them [63,65,68]. According to Reniers et al. [67], auditory, visual and/or situational signals are required to represent an individual’s cognitive and emotional state. It is important to bear in mind that empathy has a dynamic aspect that corresponds to our emotions, thoughts, and behaviours [69].

### 1.4. Cyberbullying, Parental Styles, and Empathy

Some recent research has focused on how family variables motivate adolescents to engage in cyberbullying behaviors [22,70]. Fanti et al. [71] found that communication between parents and children plays a key role in preventing online bullying, and positive communication reduces the risk of cyberbullying. In addition, in families where the relationship between members is poor and dysfunctional, there is a higher prevalence of social adjustment problems during adolescence, which increases cyberbullying practices [72]. The study by Buelga et al. [73] found that exposure to family and marital conflicts predisposes younger people to adopt antisocial, violent, and hostile behavior. According to Kowalski et al. [42], cyberbullying rates have been growing significantly due to increased internet access. Therefore, parental involvement in controlling Internet use is important for detecting and combating cyberbullying [40,41,44]. According to Martínez et al. [6], there is no clear evidence on which parental styles act as a protective or risk factor for the perpetration of cyberbullying, because the cultural and social context in which the parent–child relationship develops contributes to the variation in the behavioural adjustment of younger children. However, in some studies, it has been found that authoritarian style, due to its high punishments [5,6,72,74] and negligent style characterized by low supervision [5], are mostly related to increased cyberbullying practices.

Bronfenbrenner’s Ecological Model of Human Development [75], subsequently developed by Bronfenbrenner and Morris [76], provides a conceptual framework for understanding the perpetration of cyberbullying, on the assumption that adolescents’ behaviours result from the interaction between individual characteristics and contextual factors. Thus, the family, as the primary context of the microsystem, influences socio-emotional development, contributing to the acquisition of interpersonal skills and the regulation of behaviour in different contexts, including digital environments [47,77]. In turn, individual characteristics such as empathy have been associated with a lower tendency to engage in cyberbullying behaviour, as they promote recognition of and consideration for the consequences of aggression on the victim [78].

In addition, the family constitutes the first and most important context of socialization, capable of promoting or destroying the empathic potential of young people [77,79]. Therefore, in addition to the influence of parental styles on cyberbullying practices, it has been found that parenting plays a key role in stimulating empathy and pro-social behavior [47], with the emotional warmth of parents being able to positively predict empathic ability, and rejection by parents may have an opposite effect [79]. It is through interactions between parents and children that young people can express and clarify their feelings [61]. According to some studies, exposure to different parental styles is associated with varying levels of empathy [80,81]. However, parenting can have a distinct influence on the two components of empathy, namely cognitive and affective [82].

Some research that focused on the relationship between levels of empathy and cyberbullying points out that empathy is fundamental in managing aggressive behavior, because understanding and perceiving the emotions of others diverts the possibility of harming them [83,84,85,86]. Ratri and Andangsari [87] argue that individuals with high cognitive empathy can feel the pain and sadness of victims and are thus less likely to adopt cyberbullying behaviors. According to Salem et al. [88], empathy acts as a social anchor that prevents adolescents from engaging in unsympathetic behaviour. Therefore, lower empathy is a risk factor for cyberbullying [88,89,90,91]. According to some studies, high levels of empathy reduce aggressive behavior [84,87], i.e., it is possible to verify the existence of negative relationships between both components of empathy and the perpetration of cyberbullying [64,89,92]. However, in the study by Lazuras et al. [93], it was found that perpetrators have lower scores in affective empathy, since they tend to develop less adaptive self-efficacy beliefs and are thus more prone to the perpetration of cyberbullying.

### 1.5. Present Study

Considering what was mentioned previously, namely the vagueness about which parental style presents itself as a risk factor for cyberbullying, the lack of clear evidence about the effect that parental styles have on the two components of empathy and the differences in the levels of empathy in the perpetrators, the present study aims to: (i) analyze the association between cyberbullying behaviors and parental styles perceived by adolescents; (ii) evaluate the relationship between parental styles and the empathetic capacity of cyber bullies; (iii) analyze the association between cyberbullying and empathy; (iv) assess the differences in empathy as a function of sociodemographic variables such as gender of the participant; and (v) explore the predictive effect of parental styles and empathy on the occurrence of cyberbullying.

Based on the existing literature, the following hypotheses were formulated:

**H1.**  *Authoritarian parenting style will be positively associated with cyberbullying perpetration.*

**H2.**  *Authoritative parenting style will be negatively associated with cyberbullying perpetration.*

**H3.**  *Cognitive and affective empathy will be negatively associated with cyberbullying perpetration.*

**H4.**  *Authoritarian and authoritative parenting styles and empathy will significantly contribute to the prediction of cyberbullying perpetration.*

## 2. Materials and Methods

The present study is a descriptive, quantitative study because it aims to present results using descriptive statistics [94]. It is based on the correlational paradigm, as it is intended to evaluate the relationship between two or more variables to determine whether they covary, correlate, or exhibit some association [94]. Furthermore, it is a cross-sectional study, as observations are conducted at a single point in time [94], and it is self-report-based.

### 2.1. Participants

The final sample comprised 422 participants, from middle school and high school students in two schools in the North region of Portugal, aged between 12 and 17 years (*M* = 14.07; *DP* = 1.67), with most being female (56.6%). Only students in this age range were included. Regarding participants’ schooling, the range was from Grade 7 to Grade 12, with 18.7% in Grade 7, 32.5% in Grade 8, 23% in Grade 9, 5% in Grade 10, 8.3% in Grade 11, and 12.6% in Grade 12. Most of the participants (96.9%) are Portuguese nationals and have siblings (80.1%), with more than half of them responding that they have a sister (57.8%). In addition, 27.5% of participants live solely with both parents. Regarding the marital status of their parents, most participants come from families with married parents (78%), followed by families with divorced parents (9.5%). Regarding the type of school, a significant percentage (86.5%) replied that it is in public education. As for the age at which the Internet was first used, this ranged from 2 to 15 years (*M* = 8.65; *SD* = 2.19). In turn, more than half of adolescents spend 2 to 5 h (64.5%) daily on the Internet. Finally, 28.4% of participants primarily use YouTube.

### 2.2. Instruments

A Socio-demographic Questionnaire was used to collect the sociodemographic data of the adolescents who participated in this research, through questions related to sex, age, year of schooling, nationality, living with, marital status of parents, whether they have siblings, what type of school they attend, and issues related to the use of the Internet, such as age of start of use, daily time spent online (in hours) and the most used social network.

The Cyberbullying Questionnaire (CBQ), developed by Calvete et al. [95], was used to assess the frequency of 17 types of cyberbullying behaviour. The Portuguese version of the instrument was developed by Pinto [96]. The instrument consists of 17 items, in which participants indicate how often they performed the behaviour described in each item [96]. Response types are distributed on a Likert scale (0—Never; 1—Sometimes; 2—Often) [96]. Some items include open-ended questions for participants to describe the behaviours they practice [96]. In this questionnaire, a total score can be obtained by summing the responses to the 17 items, and high scores indicate high levels of cyberbullying behaviour [96]. In the original version of the instrument, its internal consistency was high, with a Cronbach’s alpha of 0.96 [95]. In the Portuguese version of the instrument, good internal consistency was observed with a Cronbach’s alpha of 0.91 [96].

To evaluate young people’s perceptions of the caregiving figures’ parental styles, the Parenting Styles & Dimensions Questionnaire: Short Version (PSDQ) was used. The instrument was translated and adapted for the Portuguese population by Nunes and Mota [97], from the original version by Robinson et al. [98]. This consists of 32 items, presented in two versions: one directed to the maternal figure and the other to the paternal figure [97]. Response types are distributed on a Likert scale (1—Never; 2—Sometimes; 3—Half the time; 4—Often; 5—Always) [97]. The instrument consists of three dimensions: “authoritative style “ (support and affection—5 items; regulation—5 items; giving of autonomy and democratic participation—5 items), “Authoritarian style” (physical coercion—4 items; verbal hostility—4 items; punishment—4 items) and the “Permissive style” (indulgence—5 items) [97]. Achieving superior results on each sub-scale indicates a greater perception among young people regarding the frequency of the situations described in the items [97]. In the original version, with 62 items, Cronbach’s alphas ranged from 0.91 to 0.86 for the authoritative, authoritarian, and permissive styles, respectively [98]. In the Portuguese adaptation, Cronbach’s alphas ranged from 0.86/0.81 for the full scale, 0.93/0.90 for the authoritative style, 0.76/0.80 for the authoritarian style, and 0.65/0.65 for the permissive style for paternal and maternal figures, respectively [97].

The Short Version of Basic Empathy Scale (BES-A) was used to evaluate affective and cognitive empathy in the study sample. The scale, developed by Jolliffe and Farrington [99], consists of 20 items assessing affective empathy (11 items) and cognitive empathy (9 items). In Portugal, its reduced version (BES-A) has been validated by Pechorro et al. [100]. This version comprises 7 items that assess affective empathy (3 items) and cognitive empathy (4 items) in a sample of young males and females [100]. Response types are distributed on a Likert scale (1—I totally disagree to 5—I totally agree) [100]. In this instrument, higher scores indicate greater empathy [100]. In the original version of the scale, Cronbach’s alphas of 0.85 and 0.79 were reported for the affective and cognitive empathy scales, respectively. The Portuguese version of the instrument showed good psychometric properties, with Cronbach’s alpha of 0.79 and 0.77 for the total scale, 0.79 and 0.75 for affective empathy, and 0.84 and 0.84 for cognitive empathy in males and females, respectively [100].

### 2.3. Procedure

At an initial stage, authorization was requested from the Ethics Commission of the University of Trás-os-Montes and Alto Douro (CE-UTAD). To implement the protocol in the school context, a request for authorization was made to the Directorate-General for Education (DGE), using the Monitoring of Surveys in School Environment (MIME) platform. After obtaining the DGE’s opinion, the Directorates of some Secondary Schools in the Northern region of Portugal were contacted to submit a request for authorisation to collect data at the respective institutions. After receiving the respective authorizations, the school Directors contacted the Class Directors, requesting their collaboration to deliver the Informed Consent to the students, directed to the parents in charge of education to learn about the conditions and objectives of the study and, if they wished, to authorize the students to participate in it. The protocols were then delivered to the schools, and the Directors of the institutions willing to collaborate in this investigation decided that the Class Directors should administer the questionnaires in the classroom. To preserve the sample’s heterogeneity, classes were selected at each school based on age and years of schooling.

### 2.4. Data Analysis

Given the nature of the study, the statistical analysis was performed using the Statistical Package for the Social Sciences (SPSS) (version 28). After the data were entered into the database, the sample was cleaned to exclude missing values and outliers, which were unfavorable for statistical analysis. For missing values, participants with a missing response rate greater than 10% were removed from the sample. Univariate analysis was performed using Z-scores, and multivariate analysis was performed using Mahalanobis distances. A total of 464 students were invited and gave consent; however, 42 were excluded. Therefore, 42 participants were excluded from the sample: 31 participants for missing data and 11 participants for outliers. Participants without one of the parents were excluded, thus contributing to the removed missing values. The dimensions of the instruments have also been derived from previous versions. Subsequently, normality was tested using the normality test, calculating the kurtosis (kurtosis) and skewness (asymmetry), and it was assumed that normality was met, since the sample size was large (n > 30) [101]. Then, a psychometric analysis was conducted to assess the instruments’ reliability using Cronbach’s alpha. According to Ribeiro [102], when Cronbach’s alpha exceeds 0.80, it indicates satisfactory reliability; however, values higher than 0.60 are acceptable when the instruments have fewer items.

Using the AMOS statistical program (version 28), a first-order Confirmatory Factor Analysis (CFA) was conducted to assess model fit indices.

As regards statistical analysis, parametric tests were used because they are robust to violations of the normality assumption, provided that the distributions are not significantly skewed or flattened and the sample sizes are not extremely small [103]. A significance level of 0.05 was used for the data analysis. Therefore, Pearson’s correlation was used to measure the degree of linear correlation between the quantitative variables. According to Cohen [104], correlations with values between 0.10 and 0.29 or −0.10 and −0.29 are considered low, values between 0.30 and 0.49 or −0.30 and −0.49 are considered moderate, and values between 0.50 and 1 or −0.50 and −1 are considered high. A differential analysis was carried out using multivariate analysis of variance (MANOVA), in which the partial eta-squared value was identified, which can range from 0 to 1. There is no effect of magnitude when the value is <0.01; the effect is small when the value is >0.01, moderate when it is >0.06, and strong when it is >0.14 [104]. Finally, and prior to conducting the hierarchical regression analyses, regression assumptions were examined. Hierarchical multiple regression was conducted for predictive analysis.

## 3. Results

### 3.1. Preliminary Psychometric Analyses

In the present study, in the Parenting Styles & Dimensions Questionnaire: Short Version (PSDQ), the Cronbach’s alpha coefficients were 0.86/0.83 for the full scale, 0.93/0.91 for the authoritative style, and 0.79/0.79 for the authoritarian style for the paternal and maternal figures, respectively. The permissive style yielded alpha values below 0.60 (0.52 for the father and 0.49 for the mother), so it was not used in the analysis of the present study. The results of the psychometric evaluation indicated inadequate performance of this dimension in the present sample, leading to concerns regarding its reliability and construct validity. To ensure the robustness of the following analyses, this style was not included in the final models.

The final 16-item CBQ obtained a Cronbach’s alpha coefficient of 0.89.

Finally, Cronbach’s alpha coefficients were 0.73 for the full scale, 0.78 for affective empathy, and 0.81 for cognitive empathy of the Short Version of Basic Empathy Scale (BES-A).

Subsequently, 1st-order Confirmatory Factor Analysis (CFA) was performed for all the measures using the statistical program AMOS (version 28), and the model adjustment indices were analysed. In CBQ, item 17 was deleted to obtain a satisfactory model fit, with χ^2^/df = 1.666, RMSEA = 0.04, CFI = 0.99, and NFI = 0.98. The content of item 17 appeared to capture a somewhat distinct aspect of cyberbullying behaviour compared with the remaining items, which may explain its weaker psychometric performance in the present sample.

In the Parenting Styles & Dimensions Questionnaire: Short Version (PSDQ), the permissive style was eliminated due to low alpha values. The corresponding items were removed (8, 15, 17, 20 and 24), and the following indices of adjustment were obtained for the paternal figure: χ^2^/df = 4.197; RMSEA =0.09; CFI = 0.94 and NFI = 0.93, considered to be an acceptable adjustment [105]. For the maternal figure, the indices of adjustment obtained were: χ^2^/df = 3507; *p =* 0.000; RMSEA = 0.08; CFI = 0.95 and NFI = 0.93.

Finally, for the short version of the Basic Empathy Scale (BES-A), a 1st-order Confirmatory Factor Analysis (CFA) was conducted using the AMOS statistical program (version 28), and the model fit indices were analysed. In this instrument, no changes were required, with χ^2^/df = 2.860, *p =* 0.000, RMSEA = 0.07, CFI = 0.98, and NFI = 0.96.

### 3.2. Association Between the Different Behaviors of the CBQ, the Authoritative and Authoritarian Parenting Styles of the PSDQ and the Two Components of Empathy of the BES-A

Pearson’s correlation was performed to evaluate the association between the CBQ behaviours and the parental styles of the PSDQ, namely, authoritative and authoritarian, for the father and mother figures. Next, a Pearson correlation was performed to explore the relationship between CBQ behaviors and the two components of BES-A empathy.

As for the first analysis, there were statistically significant low positive associations between CBQ and authoritarian style in the father figure (*r* = 0.231; *p* < 0.01) and mother figure (*r* = 0.228; *p* = <0.01). Based on the analyses regarding the association between cyberbullying and empathy, it was not possible to identify statistically significant associations between cyberbullying behaviors and the affective and cognitive components of empathy. The results achieved are presented in Table 1.

### 3.3. Association Between the Parental Styles of the PSDQ and the Two Components of Empathy of the BES-A

A Pearson correlation was performed to assess the relationship between the parental styles of the PSDQ and the two components of empathy in the BES-A: affective and cognitive empathy.

There were statistically significant low positive associations between the father figure’s authoritative style and cognitive empathy (*r* = 0.268; *p* < 0.01) and moderate positive associations between the mother figure’s authoritative style and cognitive empathy (*r* = 0.304; *p* < 0.01) (see Table 2).

### 3.4. Differential Analysis of the Components of Empathy of the BES-A According to the Gender of the Participant

A multivariate analysis of variance (MANOVA) was performed to analyse the differences between females and males in the two components of empathy (see Table 3).

There were statistically significant differences Wilks’ Λ = 0.949, F(2, 419) = 11.19, *p* < 0.001, *ηp*^2^ = 0.051 between males and females in the affective component of empathy [*F*(1.420) = 15.137; *p =* 0.000; *ηp*^2^ = 0.035] and in the cognitive component of empathy [*F*(1.420) = 10.409; *p* = 0.001; *ηp*^2^ = 0.024]. Analysis of estimated means indicated that female adolescents present greater affective and cognitive empathy, revealing a higher mean (Affective empathy: *M* = 3.15; *SD* = 0.96) (Cognitive empathy: *M* = 4.06; *SD* = 0.71) compared to male adolescents (Affective empathy: *M* = 2.79; *SD* = 0.90) (Cognitive empathy: *M* = 3.82; *SD* = 0.80). Based on [104] Cohen’s values (1988), the size of the effect of sex on the components of empathy is small (*ηp*^2^ ≥ 0.01).

### 3.5. Predictive Analyses: Predictive Effect of the Parental Styles of the PSDQ and the Components of Empathy of the BES-A on Cyberbullying Behaviors

A hierarchical multiple regression was performed to assess the predictive capacity of the parental styles of the PSDQ and the components of empathy of the BES-A in cyberbullying behaviors. Multicollinearity diagnostics indicated no evidence of problematic collinearity among predictors (Tolerance > 0.20; VIF < 5 for all the variables) [106].

As for Block 1, this explains 0.8% of the total variance in cyberbullying behaviours (R^2^ = 0.008), contributing individually with 0.3% of the variance to the model (R^2^ change = 0.003), and not presenting a significant contribution [F(2.419) = 1.584; *p* = 0.206]. Regarding Block 2, it makes a significant contribution [F(4.415) = 6.850; *p* = 0.000], explaining 6.9% of the total variance (R^2^ = 0.069) and contributing individually 6.1% of the variance to the model (R^2^ change = 0.061). Analysing the contribution of each independent variable in the blocks individually, we found that the authoritarian style of the father figure is a statistically significant positive predictor of cyberbullying behaviours (*β* = 0.156; *p* = 0.037; I.C. 95% [0.02; −0.83]); however, the effect size was relatively modest. Therefore, the remaining variables do not present a significant contribution (*p* > 0.05). The observed results are presented in Table 4.

## 4. Discussion

The present study aimed mainly to analyse cyberbullying from the perspective of the aggressors and their association with authoritative and authoritarian parenting styles and with the two components of empathy among adolescents attending middle and high School in schools in the North of Portugal region. More specifically, it aimed to analyze the association between cyberbullying behaviors and parental styles; assess the relationship between parental styles and the empathetic capacity of cyber bullies; analyze the association between cyberbullying and empathy; assess the differences in empathy as a function of sociodemographic variables such as the gender of the participant; and explore the predictive effect of parental styles and empathy on the occurrence of cyberbullying. Contrary to our expectations, empathy was not significantly associated with cyberbullying perpetration, and paternal authoritarian parenting style emerged as the only significant predictor in the final regression model.

In the present study, regarding the relationship between cyberbullying and the different parental styles of the PSDQ, the results revealed a positive association between perceived authoritarian style of the maternal and paternal and cyberbullying perpetration.

The same was observed in the study by Martínez et al. [6], carried out with a sample of adolescents attending public education, and it was found that young people belonging to authoritarian families tend to adopt more destructive behaviors (e.g., cyberbullying). In the study by Zurcher et al. [107], it was concluded that authoritarian behavior of parents corresponds to a potential risk factor for cyberbullying in boys, and this was not verified in girls. However, in the study by Deenamjued and Kulachai [108], no associations were found between authoritarian parental style and the practice of cyberbullying behaviors, and it was concluded that authoritarian parental behaviors are not a risk factor for perpetration. In Spain, a study by Gómez-Ortiz et al. [109] among high school students found that students from democratic families are less involved in cyberbullying. Still, young people with strict and punitive parents were the ones who reported a greater predisposition to perpetration. According to some authors, warmer parents tend to be better at alerting their children to the potential risks of internet use, thereby reducing their likelihood of cyberbullying [110,111]. Therefore, the lack of communication, attention, and the scarce involvement of parents in the activities and interests of their children, typical characteristics of the authoritarian style, seem to encourage young people to humiliate others, this being a typical reaction of adolescents who seek the recognition and attention they lack [112]. Iorga [113] in her study found that high heat and parental control contribute to decreasing the perpetration of cyberbullying. In a study by Elsaesser et al. [114], it was found that strict restrictions on access to social networks are sometimes ineffective, and that young people are less likely to engage in cyberbullying when parents collaboratively address internet issues with their children.

Regarding the relationship between cyberbullying and the two components of BES-A empathy, the results indicated no association between the variables; i.e., empathy does not appear to be associated with cyberbullying. The absence of a significant association between empathy and cyberbullying perpetration may indicate that empathy alone is insufficient to explain this form of bullying. It is possible that other variables, not explored in the present study, help to explain this association. The effects of empathy depend on other psychological and contextual factors that were not examined in the present study. Additionally, one possible explanation concerns measurement issues. The short version of the Basic Empathy Scale includes a limited number of items, which may reduce its sensitivity to detect relatively small associations. However, some studies with students have reached the same conclusion, indicating that empathy does not play an active role in cyberbullying [85,115]. Wright et al. [116] report mixed results on the relationship between cyberbullying and empathy. The investigation by Martínez et al. should be considered [117], conducted with students from different schools in the Loreto region (Amazon), which yielded contradictory and somewhat unexpected results: empathy predicts the perpetration of cyberbullying, and perpetrators show high levels of affective empathy. Steffgen et al. [118] concluded that cyberbullying abusers have less empathetic capacity than non-bullies, arguing that low levels of empathy can be a potential risk factor for perpetration, so it is necessary to invest in promoting empathy to prevent the rise in cyberbullying. In a study conducted in Singapore, individuals with low levels of affective empathy and high levels of cognitive empathy showed a lower predisposition to perpetration than those with low levels of both affective and cognitive empathy [119]. In Poland, a study was conducted with first- and second-cycle students, in which it was concluded that high affective empathy and low cognitive empathy are risk factors for the perpetration of cyberbullying [120]. In the study by Doane et al. [121], the authors concluded that boys with high levels of affective and cognitive empathy have less involvement in cyberbullying than those with low levels of cognitive empathy. Also, a study by Ratri and Andangsari [87] conducted in Indonesia found that individuals with high levels of cognitive empathy show a lower predisposition to perpetration. Therefore, the lower the empathic potential of young people, the higher the likelihood that they will engage in cyberbullying [122], since understanding others’ emotions is a fundamental skill for preventing perpetration [118].

According to the literature, parental styles have a considerable influence on the modulation of behaviors and empathic values [48,123,124]; however, it should be added that different parental styles produce distinct effects on individuals’ cognitive and affective empathy [82]. The results of the present study corroborate previous evidence, and significant positive associations were identified between the authoritative style of the paternal and maternal figures and the cognitive component of empathy. Therefore, young people from families in which a democratic style prevails have higher levels of cognitive empathy. The results are consistent with those of other studies that found that as democratic parental attitudes increase, young people’s empathic behaviours also increase [125,126,127]. This evidence may be justified by the fact that a warm family environment, in which parents support, engage with, and care about their children’s interests, helps them recognise their emotions and promote their empathic ability [124,128]. In addition, parents’ authoritative attitudes foster greater respect for others and more in-tune-with-reality ways of acting among young people [49]. In this sense, children of democratic parents tend to present higher psychosocial skills and lower deviant behaviors [129]. Specifically, regarding the authoritarian parental style, several studies indicate that it is associated with undesirable outcomes, including lower empathic capacity [123,128]. Therefore, a family environment characterized by high stiffness and severe punishment can hinder the development of empathy in individuals [127].

In the present study, significant differences were identified between females and males in both components of empathy, and female adolescents showed higher levels of affective and cognitive empathy. Although statistically significant gender differences in empathy were observed, the magnitude of the effect was relatively modest, suggesting that gender accounts for only a limited proportion of variance in empathy levels. These results are consistent with those of Baez et al. [130], who concluded that girls have higher levels of self-reported affective and cognitive empathy. Similarly, other authors have concluded that females have greater empathic capacity than males [131,132]. On the other hand, Kamas and Preston [133] did not identify gender differences in empathic capacity, suggesting that other personal characteristics shared by both sexes may be responsible for these differences. According to Muncer and Ling [134], emotional reactivity corresponds to the characteristic that produces the greatest sex differences in empathic potential. Several studies have concluded that females have higher levels of affective empathy [59,135,136]. This evidence may relate to social skills, and, culturally, females are seen as more sensitive and concerned about the interpersonal relationships they establish, seeking to respond effectively to others’ needs [130,137]. In addition, women show greater ease in recognising their emotions, which is associated with more accurate prosocial behaviour [138]. According to Stuijfzand et al. [139], sex differences in affective empathy tend to increase with the onset of adolescence due to changes in social and biological conditions typical of this developmental period. According to Jie et al. [136], male capacity for deliberation, a factor corresponding to cognitive empathy, does not differ from that of females. In addition, Olsson et al. [140] found that males show greater empathic capacity in social situations involving interactions with the opposite sex. In contrast, females exhibit greater prosocial behaviour when interacting with individuals of the same sex.

Regarding the predictive effect of affective and cognitive empathy on cyberbullying behaviours, our results indicate a non-significant contribution; i.e., the two components of empathy do not predict cyberbullying perpetration. These results are consistent with those of Mujidin et al. [85], who found that empathy does not predict cyberbullying. In the study by Graf et al. [64], affective empathy did not predict cyberbullying behaviours, whereas cognitive empathy did. In contrast, the study by Del Rey et al. [141] found that the two components of empathy predict the practice of cyberbullying behaviors in males, as well as in females, exclusively when they present low levels of affective empathy. Similarly, other studies have found that empathy is negatively associated with [82,84,122,142,143]. It should be noted that in cyberbullying, the perpetrators do not have direct contact with the victims, not knowing their suffering, which justifies their disinhibited behaviour (e.g., reduced empathy) [144]. Regarding the role of parental styles in cyberbullying behaviors, the findings provide partial support for the role of parenting in cyberbullying perpetration. Although authoritarian parenting was positively associated with cyberbullying at the correlational level, only the authoritarian parenting of the father remained a significant predictor in the final regression model. Additionally, the similarity of the bivariate correlations suggests that maternal and paternal authoritarian dimensions may share substantial variance. These results are in line with those obtained by Zurcher et al. [106], who concluded that offensive communication of the mother figure increases perpetration among boys and verbal hostility and physical coercion of the father figure contribute significantly to the adoption of cyberbullying behaviors in boys and girls. Several studies point out that authoritarian attitudes of parents predict the perpetration of cyberbullying [45,145]. On the other hand, the study by Charalampous et al. [49] concluded that authoritarian parenting has a predictive effect on aggression and victimization of traditional bullying, as well as on victimization of cyberbullying; however, the same was not verified in the perpetration of cyberbullying. Young people whose relationships with their parents are difficult and problematic, where a lack of communication prevails, show a greater tendency to use online resources and consequently practice cyberbullying [112]. Therefore, according to the literature, these findings suggest that, in this sample, an authoritarian parental style may represent a risk factor for cyberbullying perpetration, although this relationship should be interpreted within the sociocultural context of the study and requires confirmation in other populations [45], while an authoritative parental style is a protective factor against cyberbullying [146]. Although the final regression model was statistically significant, it explained only 6.9% of the variance in cyberbullying perpetration; the results should be analyzed with caution. This finding suggests that authoritative and authoritarian parenting styles and empathy represent only a small part of the factors associated with cyberbullying involvement. Cyberbullying is widely recognized as a multifactorial phenomenon influenced by individual characteristics, contextual factors (e.g., peer relationships, school climate, online experiences, moral disengagement) and other factors not analyzed in the present study.

### 4.1. Practical Implications

The main objective of this study was to analyze the phenomenon of cyberbullying from the perspective of the aggressors and their association with the authoritative and authoritarian parenting styles and with the two components of empathy in a sample of Portuguese adolescents. It should be noted that few studies analyze the effect of parental styles and their particularities on adolescents’ online activities; however, studies conducted to date confirm the influence of certain parental styles on the increase in cyberbullying perpetration. The same is true for the effect of empathy on cyberbullying behaviors, and most existing studies support this association.

In this study, the results obtained offer an increase in knowledge about the relationships between parental styles, empathic ability, and cyberbullying behaviors. Although the observed effects were modest, the findings suggest that parenting practices, particularly authoritarian parenting, may represent one of several factors associated with cyberbullying perpetration. That is, it may be relevant to develop programs to educate parents on how to interact positively with their children. In addition, the programs should include information on the importance of pro-social behavior and its contribution to building more harmonious relationships. It may also be beneficial to develop awareness-raising activities for adolescents, parents, teachers, and other caregivers to highlight the consequences of Internet misuse. Therefore, to reduce cyberbullying, there should be investment from young people, families, and schools, as well as special attention to changing parental practices [106]. In addition, to protect young people from cyberbullying and other anti-social behavior, it is necessary to promote empathy, which can be done through school-based campaigns [132]. However, recommendations for interventions focused on empathy should be considered with caution, as empathy did not significantly predict cyberbullying perpetration in the present study. The same caution should be applied to parent-focused interventions. Although paternal authoritarian parenting style was significantly associated with cyberbullying perpetration, the cross-sectional nature of the study does not permit causal conclusions.

### 4.2. Limitations

Several limitations should be considered when interpreting the findings of this study.

First, all the variables, including parenting styles, empathy, and cyberbullying perpetration, were assessed using adolescent self-report measures. Collecting all data from the same respondents at a single point in time may have increased the risk of common method variance and social desirability bias, particularly given the sensitive nature of cyberbullying behaviours. Participants may have underreported their involvement or have concerns about presenting themselves in a favourable light. In addition, adolescents’ reports reflect their perceptions of parenting practices, which may not fully correspond to parents’ own behaviours. Furthermore, the absence of parent-, teacher-, or peer-report data prevented triangulation of information across multiple informants, potentially reducing the robustness of the findings. As such, the strength of some observed associations should be interpreted with appropriate caution.

Second, the cross-sectional design limits the conclusions regarding the direction of the observed relationships. Although the findings suggest associations between parenting styles and cyberbullying, causal inferences cannot be drawn, and it is possible that these relationships are reciprocal or influenced by other factors not assessed in the present study.

The sample was drawn from schools in the northern region of Portugal, using a non-probabilistic sampling strategy, which may limit the generalisability of the findings to adolescents from other regions or cultural contexts. Replication in more diverse samples would help determine the extent to which these findings are consistent across different populations.

Although the short version of the Basic Empathy Scale demonstrated acceptable reliability and validity, its reduced number of items may not have captured the complexity of the empathy construct as comprehensively as the full version. Consequently, subtle associations between empathy and cyberbullying may have been more difficult to detect, which may partly explain the absence of significant relationships observed in the present study.

Another consideration is that the predictive model explained a relatively small proportion of the variance in cyberbullying behaviours. This suggests that, while parenting styles appear to play a role, cyberbullying is likely influenced by a broader range of individual, family, peer, and contextual factors that were beyond the scope of the present study. In addition, potentially relevant variables, such as age, gender, family structure, patterns of internet use, cybervictimization, traditional bullying, and bystander behaviour, were not included in the regression model and may also contribute to explaining cyberbullying perpetration. Furthermore, although participants were recruited from different schools, the analyses did not account for the potential clustering of students within schools or classrooms, which may have influenced the precision of the estimated associations.

Additionally, the use of multiple statistical tests may have increased the risk of Type I error. The findings should nevertheless be interpreted with caution and replicated in future studies. Another statistical limitation was that the CFA modifications and item deletions were performed in the same sample used for hypothesis testing, so the risk of overfitting should be acknowledged.

Another limitation is that the invariance of the measurement between groups was not evaluated. Therefore, the differences observed between the sexes should be interpreted with caution, as the equivalence of the measurement model between groups has not been established.

Finally, the permissive parenting style was not included in the analyses due to its low internal consistency in the instrument’s Portuguese version. Although this decision was made to ensure the robustness of the analyses, it meant that the full range of parenting styles could not be examined. In addition, although negligent parenting was discussed in light of the existing literature, this parenting dimension was not directly assessed in the present study and therefore could not be empirically evaluated. Likewise, while the brief version of the empathy scale demonstrated acceptable psychometric properties, a more comprehensive assessment of empathy may provide additional insight into its relationship with cyberbullying.

### 4.3. Proposals for Future Studies

Future research would benefit from longitudinal, multi-informant designs and the inclusion of a broader range of contextual variables to further clarify the relationships identified in the present study. Future studies would benefit from incorporating alternative assessment methods to reduce the potential influence of self-report bias. Also, regarding the measures, it is recommended that future studies use more comprehensive measures of empathy to further examine these relationships and determine whether different dimensions of empathy are differentially associated with cyberbullying perpetration. Future research should also explore the potential role of variables such as gender, moral disengagement, emotion regulation, peer processes, and online interaction characteristics in explaining the relationship between empathy and cyberbullying perpetration. The CFA modifications and item deletions were performed in the same sample used for hypothesis testing, so the risk of overfitting should be acknowledged. Future studies should replicate the factor structure of the Cyberbullying Questionnaire in independent samples.

Although the present study focused on examining the associations between parenting styles, empathy, and cyberbullying perpetration, future research could extend these findings by employing alternative analytical approaches. For example, statistical models that adjust for relevant covariates (e.g., socioeconomic status, family structure, or internet use) and account for the clustering of participants within schools may provide a more comprehensive understanding of the relationships among these variables and strengthen the robustness of the findings.

## Figures and Tables

**Table 1:** Correlations between the Behaviors of CBQ, the Parental Styles of the PSDQ and the Empathy Components of BES-A, Mean and Standard Deviation (N = 422).

		1	*M ± SD*
CBQ		-	0.07 ± 0.18
PSDQ			
Father	Authoritative Style	−0.034	3.58 ± 0.95
	Authoritarian Style	0.231 **	2.03 ± 0.64
Mother	Authoritative Style	−0.027	3.79 ± 0.83
	Authoritarian Style	0.228 **	2.06 ± 0.63
BES-A	Affective Empathy	0.052	3.0 ± 0.95
	Cognitive Empathy	−0.059	3.96 ± 0.76

Note: CBQ = Cyberbullying Questionnaire; PSDQ = Parenting Styles & Dimensions Questionnaire; BES-A = Short Version of Basic Empathy Scale; *M* = Mean; *SD* = Standard Deviation; ** *p* < 0.01.

**Table 2:** Correlations between Parental Styles of PSDQ and Empathy Components of BES-A, Mean and Standard Deviation (N = 422).

	1	2	*M ± SD*
BES-A			
Affective Empathy	-	-	2.99 ± 0.95
Cognitive Empathy	-	-	3.96 ± 0.76
PSDQ			
Authoritative Style (Father)	0.012	0.268 **	3.58 ± 0.95
Authoritarian Style (Father)	−0.057	0.037	2.03 ± 0.64
Authoritative Style (Mother)	0.047	0.304 **	3.79 ± 0.83
Authoritarian Style (Mother)	−0.005	−0.024	2.06 ± 0.63

Note: BES-A = Short Version of Basic Empathy Scale; PSDQ = Parenting Styles & Dimensions Questionnaire; *M* = Mean; *SD* = Standard Deviation; ** *p* < 0.01.

**Table 3:** Differential Analysis of the Components of Empathy of the BES-A as a Function of the Participant’s Sex.

BES-A	Gender	*M ± SD*	Direction of Difference
Affective Empathy	Female	3.15 ± 0.96	1 > 2
	Male	2.79 ± 0.90	
Cognitive Empathy	Female	4.06 ± 0.71	1 > 2
	Male	3.82 ± 0.80	

Note: BES-A = Short Version of Basic Empathy Scale; *M* = Mean; *SD* = Standard Deviation.

**Table 4:** Predictor Role of Parental Styles of PSDQ and Components of Empathy of the BES-A in Cyberbullying Behaviors.

		R^2^	R^2^ Change	ΔF	B	*SE*	*β*	*t*	*p*	IC95%
CBQ										
Block 1	BES-A	0.008	0.008	1.584						
	Affective Empathy				0.012	0.009	0.064	1.294	0.196	[−0.004–0.006]
	Cognitive Empathy				−0.016	0.011	−0.070	−1.421	0.156	[−0.040–0.006]
Block 2	PSDQ	0.069	0.061	6.848						
	Authoritative Style (Father)				−0.001	0.016	−0.003	−0.036	0.971	[−0.031–0.030]
	Authoritarian Style (Father)				0.043	0.020	0.156	2.089	0.037	[0.002–0.083]
	Authoritative Style (Mother)				0.000	0.018	0.001	0.017	0.987	[−0.035–0.036]
	Authoritarian Style (Mother)				0.030	0.021	0.108	1.464	0.144	[−0.010–0.071]

Note: CBQ = Cyberbullying Questionnaire; BES-A = Short Version of Basic Empathy Scale; PSDQ = Parenting Styles & Dimensions Questionnaire.

## Data Availability

The data presented in this study are available upon request from the corresponding author. The data are not publicly available due to privacy and ethical reasons.
